# Impact of Alcohol on Glycemic Control and Insulin Action

**DOI:** 10.3390/biom5042223

**Published:** 2015-09-29

**Authors:** Jennifer L. Steiner, Kristen T. Crowell, Charles H. Lang

**Affiliations:** 1 Department of Cellular and Molecular Physiology, Penn State College of Medicine, Hershey, PA 17033, USA; E-Mail: jls1075@psu.edu; 2 Department of Surgery, Penn State College of Medicine, Hershey, PA 17033, USA; E-Mail: kcrowell1@hmc.psu.edu

**Keywords:** ethanol, skeletal muscle, liver, insulin action, alcohol use disorder

## Abstract

Alcohol has profound effects on tissue and whole-body fuel metabolism which contribute to the increased morbidity and mortality in individuals with alcohol use disorder. This review focuses on the glucose metabolic effects of alcohol, primarily in the muscle, liver and adipose tissue, under basal postabsorptive conditions and in response to insulin stimulation. While there is a relatively extensive literature in this area, results are often discordant and extrapolating between models and tissues is fraught with uncertainty. Comparisons between data generated in experimental cell and animals systems will be contrasted with that obtained from human subjects as often times results differ. Further, the nutritional status is also an important component of the sometimes divergent findings pertaining to the effects of alcohol on the regulation of insulin and glucose metabolism. This work is relevant as the contribution of alcohol intake to the development or exacerbation of type 2 diabetes remains ill-defined and a multi-systems approach is likely needed as both alcohol and diabetes affect multiple targets within the body.

## 1. Introduction

Glucose homeostasis is critical for normal functioning of the central nervous system and cells which have an obligatory requirement for this metabolic substrate. Acute and chronic alterations in the prevailing glucose concentration (*i.e.*, hypoglycemia and hyperglycemia) can adversely impact cellular and organ function. This review focuses on the etiology of ethanol (*i.e*., alcohol)-induced changes in glycemic control and insulin action at the molecular, cellular and tissue level, integrating the response of key glucoregulatory tissues including skeletal and cardiac muscle, adipose tissue and liver. As the underlying mechanisms of alcohol-induced changes are oftentimes dependent on the exposure time and intoxication level, these variables will be identified and accounted for when relevant. We will narrow our discussion to the effects of insulin on carbohydrate metabolism, but certainly acknowledge the potent metabolic effects this hormone has on both lipid and protein metabolism as well as the effect of alcohol on the secretion of other hormones [1]. Also, the literature pertaining to the ability of alcohol to alter insulin signaling pertaining to metabolic processes other than carbohydrate metabolism (e.g., hepatocyte growth and survival) will not be reviewed due to its extensive nature and readers are referred to other work on this topic [2]. Lastly, there is an equally extensive collection of literature on the effects of alcohol in individuals with type I and type II (±obesity) diabetes and it is not possible to include a systematic review of this topic. Throughout, where possible, we have highlighted limitations of various approaches which may complicate data interpretation and provide suggestions for future research opportunities in this area.

At least half of US adults consume alcohol and as many as 5% can be categorized as heavy drinkers (*i.e*., men > 14 standard drinks/day; women > 6 standard drinks/day), with the number in the latter category increasing over the past decade [3,4]. Alcohol has also been reported to have the greatest combined harm to society as a whole, compared to recreational drugs, when the cost to the individual, family and community is collectively considered (e.g., multiple-criteria decision analysis) [5]. This review aims to provide a better understanding of the intricacies and integrative nature of cellular and molecular mechanisms by which acute and chronic alcohol abuse regulates a one key element of whole-body metabolic control—glucose homeostasis.

## 2. Alcohol and Basal Glucose Homeostasis

### 2.1. Blood Glucose Concentrations

Studies in humans and a variety of preclinical models indicate that acute administration of alcohol can lead to either a reduction or no change in the circulating concentration of glucose. This dichotomy can be largely explained by the nutritional state of the host at the time alcohol is administered (e.g., duration of the fast or lack thereof), the amount of alcohol administered and the resulting blood alcohol level (BAL). For example, in humans fasted ~12 h (*i.e*., overnight), alcohol does not typically alter the blood glucose concentration [6,7,8,9,10,11]. Likewise, euglycemia is maintained in overnight fasted rats [12] and mice [13] after acute alcohol intoxication. Moreover, there is little evidence of acute alcohol-induced hypoglycemia in humans or animals under the more physiological relevant condition of adequate nutrition [14,15,16]. In contrast, a severe and sustained hypoglycemia is elicited when alcohol is acutely administered to humans [9,17,18] or animals [19,20] fasted ~3–4 days. Thus, hypoglycemia would only be anticipated in humans with alcohol use disorder (AUD) who also have a relatively poor nutritional status or severely impaired liver function [21]. This conclusion is supported by a recently published meta-analysis of intervention studies in humans which concluded there was no effect of alcohol consumption (10–70 kg/day) on the glucose concentration in nondiabetic individuals [22], which corroborated findings from several earlier large prospective, cross sectional population studies [23,24].

### 2.2. Whole-Body Basal Glucose Flux

The prevailing blood glucose concentration is representative of discrete metabolic processes which regulate the rate of appearance (Ra) for glucose *versus* those which consume and regulate glucose disappearance (Rd). Glucose Ra represents glucose influx into the circulation primarily from the liver via glycogenolysis and gluconeogenesis as well as nutrient absorption from the gastrointestinal tract, with the relative contribution of each source determined by the nutritional state of the host and the experimental circumstances. As few metabolic studies are performed in the fed condition, contribution of glucose from gastrointestinal tract absorption to whole-body glucose Ra is typically considered be nominal after an overnight (or longer) fast. Further, despite the possibility that alcohol may increase intestinal glucose absorption, any alcohol-induced change in whole-body glucose Ra is primarily considered a manifestation of glucose output by the liver [25,26].

Isotope dilution methodology has provided a more thorough understanding of the whole-body glucose carbon flux in response to alcohol. For example, in experimental conditions where acute alcohol produced hypoglycemia (*i.e*., prolonged fast), there is a consistent and profound decrease in glucose Ra accompanied by an inappropriately elevated rate of glucose Rd [17,19,20]. In contrast, under nutritional conditions favoring maintenance of euglycemia, acute alcohol results in either no change in whole-body glucose flux [11,27,28,29] or a decrease in glucose Ra which is offset by a proportional reduction in glucose Rd [6,29,30]. Similarly, whole-body glucose Ra and Rd do not differ under conditions where alcohol is chronically consumed by rats [14,28] or humans [6].

### 2.3. Basal Hepatic Glucose Metabolism

*In vivo* determination of transhepatic glucose flux in 48–72 h fasted dogs, with essentially no glycogen reserves, indicates acute alcohol markedly impairs gluconeogenesis [31]. Alcohol also dose-dependently inhibits lactate-stimulated gluconeogenesis when given acutely in the *in situ* perfused liver [32] and when added to isolated hepatocytes [33]. While this ability of alcohol to antagonize the effective utilization of lactate partially explains the hyperlactacidemia observed after acute alcohol in both short- and long-term fasted subjects [10,20,27], alcohol also increases net lactate output from skeletal muscle [34]. Collectively, these data are consistent with those from *in vivo* studies showing acute alcohol decreases whole-body estimates of glucose recycling (e.g., glucose → lactate → glucose) and lactate turnover [27].

With respect to the other major gluconeogenic substrates, alcohol acutely impairs the *de novo* synthesis of glucose from glycerol both *in vivo* [34,35] and *in vitro* [32,36] and from alanine in a dose-dependent manner [32,37]. In contrast, gluconeogenesis from pyruvate is unaltered or even elevated by acute alcohol [38,39]. These metabolic effects are a consequence of the oxidative metabolism of alcohol via alcohol dehydrogenase which increases the NADH/NAD^+^ ratio and thereby reduces the pyruvate/lactate ratio to inhibit hepatic gluconeogenesis [31,32,40]. Accordingly, pretreatment with a specific inhibitor of alcohol dehydrogenase, 4-methylpyrazole, prevents the alcohol-induced inhibition of gluconeogenesis [38]. Additionally, alcohol has been reported to effect hepatic glycolytic and gluconeogenic enzyme activities including a reduction in phosphofructokinase [41]. The resulting alcohol-induced decrease in pyruvate lowers pyruvate carboxylase, the rate limiting gluconeogenic enzyme, thereby contributing to the decrease in hepatic glucose output [38]. Aside from these changes, however, reports are inconsistent and contradictory regarding the effects of alcohol on glycolytic and gluconeogenic enzymes [33,38,42].

In contrast to the numerous studies on the acute effects of alcohol on hepatic gluconeogenesis, only two studies have examined *de novo* glucose production by liver from animals chronically consuming an alcohol-containing diet. In this regard, hepatocytes isolated from chronic alcohol-fed rats had lower rates of lactate-derived gluconeogenesis [43] and the gluconeogenic capacity of *ex vivo* perfused liver from female alcohol-fed rats was reduced [44].

As noted above, alcohol administered acutely does not generally decrease whole-body glucose Ra but does markedly suppress gluconeogenesis. This metabolic scenario is consistent with alcohol acutely stimulating hepatic glycogenolysis under fed or short-term fasted conditions to defend against the development of hypoglycemia [9,11,45,46,47]. Many studies indicate that chronic alcohol intake decreases whole liver glycogen content [47,48,49] which occurs in both periportal and perivenous hepatocytes [50], and is consistent with the observed reduction in basal glycogen synthase activity. The decrease in hepatic glycogen appears to result from the ability of alcohol to inhibit the repletion of glycogen reserves which is directly related to the concomitant inhibition of gluconeogenesis [9,46,49].

Although many of the human studies described above included men and women, none had sufficient statistical power to discern the presence of any sexual dimorphic response of alcohol on whole-body glucose flux. Likewise, essentially all of the preclinical studies used male animals. Only the work by Sumida *et al.* [29] has attempted to systematically investigate sex differences in this regard and their work suggests that alcohol has a more pronounced inhibitory effect on hepatic gluconeogenesis in chronic alcohol-fed female rats.

### 2.4. Basal Glucose Disposal by Muscle and Peripheral Tissues

#### 2.4.1. Glucose Uptake by Striated Muscle

Although most studies indicate that acute and chronic alcohol intake does not dramatically change total whole-body glucose disposal under basal conditions, such measurements assess the integrated effect of alcohol on numerous peripheral tissues. Thus, studies have also determined whether alcohol might alter glucose uptake in a tissue-specific manner. As a metabolically active tissue representing 40%–45% of total body weight, skeletal muscle has been the focus of many of these studies. However, in large part, the data are equivocal in nature. For example, Molina *et al.* [51] used an *in vivo* injection of ^14^C-radiolabeled 2-deoxyglucose (2DG) to trace regional glucose uptake in rats during a 4 h continuous infusion of alcohol that did not alter basal glucose or insulin concentrations. In response to alcohol, glucose uptake in the gastrocnemius was reduced while no change was observed in the white or red quadriceps, abdominal muscle or diaphragm. Using a similar model of acute alcohol administration, Spolarics *et al.* [12] also reported reduced glucose uptake in some muscles (e.g., red quadriceps and soleus), but not others (e.g., gastrocnemius and white quadriceps). These results are mostly consistent with data from early arterio (A)—venous (V) difference studies in humans demonstrating alcohol acutely inhibits basal muscle glucose disposal [34,52], although they differ from others that showed acute alcohol increased muscle glucose uptake [53,54]. The reason for these discrepant findings in humans is not readily apparent as alcohol dose, methodology and subject demographics were comparable. Finally, basal glucose uptake is unchanged in primary cultured rat skeletal muscle acutely incubated with up to 100 mM ethanol for up to 24 h [55,56].

Basal *in vivo*-determined glucose disposal by skeletal muscle, both fast- and slow-twitch fiber types, also did not differ between control and chronic alcohol-fed rats [14]. Similarly, *in vitro*-determined basal glucose uptake did not differ in incubated epitrochlearis muscle isolated from pair-fed and alcohol-fed rats [57]. The differences in muscle glucose uptake between acute and chronic alcohol exposure has been posited to be due to the relatively lower peak BAL achieved in chronic alcohol-fed rats. These data and others presented below suggest that acute ethanol intoxication may not accurately reflect the new metabolic steady-state achieved in during chronic intake. Lastly, it is noteworthy that despite the unaltered glucose uptake by skeletal muscle from alcohol-fed rats, a decrease in the phosphorylation of the insulin receptor, insulin receptor substrate (IRS)-1 and AKT has been observed under basal non-insulin stimulated conditions in some studies [58] but not others [14]. Such differences highlight the importance of including physiologically relevant endpoints in studies focusing on signal transduction. Collectively, these relatively divergent data fail to provide convincing evidence for a consistent inhibitory effect of either acute or chronic alcohol exposure on basal glucose disposal by skeletal muscle.

Even under basal postabsorptive conditions, glucose disposal in skeletal muscle (as well as in heart and adipose tissue) occurs by two mechanisms—insulin-mediated glucose uptake (IMGU) and noninsulin-mediated glucose uptake (NIMGU). While, as the name implies, insulin facilitates glucose disposal via the first mechanism, NIMGU is driven primary by the mass action effect of glucose and accounts for ~75%–80% of basal whole-body glucose disposal [59]. Hence, some of the discordance noted above related to the alcohol-induced changes in muscle glucose uptake may be attributable to between-study differences in the prevailing concentrations of glucose, even if such changes fail to achieve statistical significance. Low-dose alcohol does not acutely alter whole-body glucose effectiveness [60,61] but there are no data pertaining to the ability of alcohol to independently regulate NIMGU at the tissue level.

Basal glycogen content in skeletal muscle has most often been reported to be unaltered by chronic alcohol ingestion [62,63], but some studies have shown elevated glycogen content [64] in the absence of overt symptoms of alcoholic myopathy. Neither acute alcohol intoxication [25] nor chronic alcohol feeding for 6 weeks in rats alters basal muscle glycogen content [65], despite the ability of acute alcohol to antagonize glucose-stimulated glycogen repletion in skeletal muscle [66].

#### 2.4.2. Glucose Uptake by Heart

Using similar animal models and methodological approaches as described in the preceding section, acute alcohol either does not change or decreases basal *in vivo*-determined cardiac glucose uptake [12,51]. Basal cardiac glucose uptake (both atria and ventricle) also did not differ between pair-fed and chronic alcohol-fed rats [14]. While these findings are consistent with the lack of change in the total amount and phosphorylation of insulin receptor and IRS-1 in hearts of control and alcohol-fed rats [67,68], other studies report a dose-dependent alcohol-induced decrease in these early steps in the insulin signal transduction pathway in cardiac muscle [69]. Finally, short-term (30 min) culture of cardiomyocytes (H9c2 cells) with alcohol (50 mM) increases glucose uptake in association with an increase in translocation of GLUT4 from the cytosol to the plasma membrane [70]; however, the short time frame in which glucose metabolism was assessed may limit the relevance of these data. Similar to skeletal muscle, the data pertaining to the effect of alcohol on basal glucose uptake in heart does not present a consistent picture as to an underlying defect, which may be a consequence of differences in animal models and methodology.

#### 2.4.3. Glucose Uptake by Adipose Tissue

Different from the divergent reports for muscle, data consistently show that neither acute nor chronic alcohol impairs basal glucose uptake by adipose tissue determined both *in vivo* [12,14,71] and *in vitro* in isolated adipocytes [72]. Moreover, the lack of an alcohol effect on basal glucose uptake was observed in both perirenal and epididymal fat deposits [14]. These findings are consistent with the lack of an alcohol-induced change in the major transporter responsible for noninsulin-independent glucose transport in fat, GLUT1 [72].

#### 2.4.4. Glucose Uptake by other Peripheral Tissues

Evidence of an alcohol effect on glucose uptake by other peripheral tissues is limited. It appears that neither acute alcohol intoxication nor chronic alcohol feeding consistently alters basal glucose uptake by skin, intestine, spleen, lung, kidney or whole liver [12,14,73]. Further, alcohol did not alter *in vivo* glucose uptake by hepatocytes, Kupffer cells or hepatic endothelial cells [74]. These findings are divergent to that observed in other catabolic conditions where glucose uptake is enhanced in macrophage-rich tissues [75].

In contrast to the limited data available on alcohol-induced changes in glucose uptake for most peripheral tissues, there is a considerable body of literature pertaining to glucose uptake by whole brain and by various brain regions. In human volunteers, acute alcohol decreased the glucose arterial-jugular vein difference suggesting a reduction in total brain glucose uptake [76]. Similarly, an early study using PET imaging in humans also reported a reduction in brain glucose uptake after acute alcohol intoxication [77]. Likewise, rodent studies show acute alcohol-induced inhibition of glucose uptake in several brain regions [78,79,80,81] and a decrease in the rate of glucose utilization by numerous regions in the isolated perfused mouse brain [82]. However, in recent studies the *in vivo* uptake of 2-DG by the whole brain did not differ in response to either acute [12,73,83] or chronic [14] alcohol in rats. Despite these recent conflicting observations, most data from human and preclinical studies suggest that alcohol decreases basal glucose uptake by the brain.

## 3. Alcohol-Induced Changes in Basal Insulin and Glucose Tolerance

### 3.1. Glucose Tolerance

The effect of alcohol on glucose tolerance in nondiabetic subjects and animals is often contradictory making data interpretation problematic. In general, glucose tolerance has been reported to impaired, improved and unaffected by alcohol, as described below. These discordant findings highlight the equivocal nature of the data obtained from standard glucose tolerance tests (GTTs), especially when concomitant insulin concentrations over time are not also provided. In particular, while GTTs appear to primarily reflect peripheral insulin sensitivity, they also contain a component of noninsulin-mediated glucose disposal or glucose effectiveness. Additionally, all studies using an oral glucose challenge have some inherent limitations. The first is related to the possibility that alcohol can decrease gastric motility and emptying which may inhibit glucose absorption [25]. The second pertains to the effect of alcohol on glucose-stimulated secretion of gastrointestinal hormones (e.g., incretins) which can impact insulin secretion and/or glucose disposal [84]. Despite these recognized limitations, GTTs are still routinely performed because of their simplicity and the results often erroneously used to imply changes in insulin action.

#### 3.1.1. No Change in Glucose Tolerance

Both the relatively short-term (several hours) infusion of alcohol (10–20 mM) [85,86] as well as longer-term 1–3 weeks of moderate alcohol consumption did not alter glucose tolerance, as assessed by the area under the curve (AUC) for glucose after a standard oral glucose tolerance test (OGTT) in otherwise healthy nonobese humans [61,87,88]. Likewise, there was no change in glucose tolerance in chronic alcohol-fed rats [89,90,91] or mice [92].

#### 3.1.2. Improved Glucose Tolerance

Oral consumption of a moderate dose of alcohol at various times preceding an OGTT in humans has also been reported to improve glucose tolerance, which in this case may have resulted from an increase in pancreatic insulin secretion [26,93]. Similarly, chronic alcohol-fed mice have been reported to have improved glucose tolerance [94]. Some of this apparent discrepancy between these studies and the ones discussed in previous sections may be explained by a biphasic dose response (inverted U-shaped curve) to alcohol [95]. In this study an intravenous (IV) GTT was performed on rats maintained on different percentages of alcohol in drinking water. The data herein show that both relatively low (1%–2%) and high (7%) amounts of alcohol do not alter glucose disappearance, but that moderate doses of alcohol (3%) increase glucose tolerance and reduce the AUC for glucose. Large population-based studies have also reported that moderate alcohol intake over many years (>10 years) improves glucose tolerance and reduces the glucose-induced insulin secretion [96], implying increased insulin sensitivity.

#### 3.1.3. Impaired Glucose Tolerance

Other studies in humans who have chronically consumed alcohol (>20 years), but do not present with hepatic steatosis or cirrhosis, report impaired glucose tolerance following an oral GTT [16,97]. Impaired tolerance was also detected in humans and rats after a single oral ingestion of alcohol [98,99] as well as in chronic alcohol-fed rats and mice [13,57,100]. Furthermore, alcohol-induced glucose intolerance was exaggerated in transgenic mice overexpressing cardiac-specific alcohol dehydrogenase [13], suggesting this alcohol effect is mediated directly as opposed to one of its metabolites.

### 3.2. Insulin Secretion and Plasma Concentrations

The strong consensus from *in vitro* and *ex vivo* models, although not entirely consistent, suggests that alcohol inhibits insulin secretion. Using the isolated perfused pancreas, alcohol did not alter basal insulin secretion but did impair glucose-stimulated insulin secretion (GSIS) in a dose-dependent manner [101]. Other studies reported that alcohol inhibits both early- and late-phase insulin secretion by the perfused rat pancreas [101,102]. Acute *in vitro* treatment with alcohol or its metabolite, acetaldehyde, also dose-dependently reduces GSIS in isolated islets [103]. Moreover, a similar alcohol-induced reduction was observed when alcohol was administered *in vivo* and islet insulin secretion was assessed *in vitro* [104]. Likewise, incubation of INS-1 cells with 60 mM alcohol acutely reduced basal insulin secretion in a gamma-aminobutyric acid (GABA)-dependent manner [105]. In one of the most thorough *in vitro* examination of the effect of alcohol on insulin secretion, alcohol had a dose-dependent inhibitory effect on basal and GSIS in INS-1 cells, dependent in part upon the duration of cell exposure to alcohol [106]. This inhibitory effect resulted from impaired muscarinic signaling and PKC activation, but was K-ATP channel-independent. Lastly, basal and GSIS are decreased in isolated islets from chronic alcohol-fed mice [100]. Thus, alcohol and its metabolites appear to have a consistent inhibitory effect on GSIS under *in vitro* conditions.

It might be anticipated based on the above-mentioned *in vitro* data that the acute *in vivo* administration of alcohol would decrease the circulating insulin concentration. While there are scattered reports of relatively mild alcohol-induced hypoinsulinemia [23], the majority of studies show basal postabsorptive plasma insulin concentrations do not differ significantly from control values [85,107,108]. For example, neither a single oral dose of alcohol [93] nor a 4 h intravenous infusion altered plasma insulin concentrations determined 12 h later. Similarly, no change in the plasma insulin concentration was reported in chronic alcohol-fed rats [14,57], which is consistent with the lack of a significant change in pancreatic insulin content [89]. Additionally, 1–3 weeks of moderate alcohol consumption in humans did not alter the basal insulin concentration [61,87] and plasma insulin did not differ after long-term moderate alcohol intake [96]. The relationship between the magnitude of alcohol consumption and basal insulin concentrations may also be J- or U-shaped. For example, mild to moderate alcohol consumption in humans has been repeated demonstrated to decrease fasting insulin levels relatively to subjects consuming no/low alcohol and/or those with a high alcohol intake [22,23,24,109,110].

In contrast to its effect on insulin secretion *in vitro* and *ex vivo*, alcohol administered *in vivo* has been shown to enhance GSIS; in some instances this potentiated secretion is sufficient to increase the rate of glucose clearance from circulation (e.g., improve glucose tolerance) [26,107], but in others glucose tolerance remains impaired [99,111]. This priming effect develops within several hours [108] and occurs at relatively low alcohol concentrations (10 mM) [85]. Moreover, the ability of alcohol to enhance insulin secretion in humans was maintained in response to repetitive glucose challenges given over a 2 h period [93]. Such a priming effect, however, has not been observed in rats either after acute alcohol administration [98] or chronic alcohol feeding [57], but alcohol did inhibit the stimulatory action of the insulin secretagogue tolbutamide [98].

Although first-phase insulin secretion (obtained from a frequently-sampled IVGTT) was decreased in a dose-dependent manner in alcohol-consuming humans [61], interpretation of data from this study is complicated by time-dependent fluctuations in both plasma glucose and insulin. To circumvent this concern, a variable infusion of glucose was administered, which effectively clamped the blood glucose at either fed or high physiological concentrations during the concomitant infusion of saline or alcohol. Under this well-controlled condition, individuals infused with alcohol showed potentiation of both the early- and late-phase release of insulin [86]. Alcohol-induced differences in plasma insulin appear independent of a change in hepatic insulin extraction [85]. Finally, the priming effect in humans appears specific to glucose as it does not impair glucagon-mediated insulin secretion [93].

Complicating the extrapolation of such data are several reports in which short-term (1–10 week) moderate alcohol consumption in humans [87,88,112] or chronic alcohol feeding in rats [89] did not alter GSIS over the 2 h time course of the standard oral or intravenous GTT. Furthermore, acute alcohol administration [98] and long-term ingestion of moderate doses of alcohol consumption [113] have been infrequently reported to reduce insulin secretion. Hence, while alcohol appears to inhibit insulin secretion *in vitro*, its effects under *in vivo* conditions are more variable and may be model- and/or species-dependent.

## 4. Impact of Alcohol on Insulin Action

### 4.1. Whole-Body Insulin Resistance

Data from the standard glucose tolerance test provides information on glucose tolerance, but is a poor predictor of insulin action/resistance especially in the absence of accompanying insulin levels [114]. In this regard, the euglycemic hyperinsulinemic clamp is considered the gold standard and is extensively used to directly assess whole-body insulin action. In response to acute alcohol administration, numerous studies have demonstrated the presence of whole-body insulin resistance, as inferred by the lower exogenous glucose infusion rate (GIR) necessary to maintain euglycemia or the GIR/insulin ratio. Although there is one report that acute alcohol enhances insulin action [86], the majority of studies in healthy humans show that alcohol acutely decreases whole-body insulin-stimulated glucose uptake when the plasma insulin concentration is raised to high physiological levels indicative of the fed state [7,30,52,115]. Further, in one study, alcohol produced a right-shift in the insulin dose-response curve suggesting both a decrease in insulin sensitivity and maximal responsiveness [115].

Acute alcohol intoxication also produces whole-body insulin resistance in rats [12,116] and the alcohol effect appears to be dose-dependent [117,118,119]. As the alcohol-induced impairment was recapitulated by *t*-butanol (a non-metabolizable alcohol) and not antagonized by 4-methylpyrazole, the insulin resistance was likely mediated by alcohol and not one of its oxidative metabolites [117]. Furthermore, numerous studies have also demonstrated impaired whole-body IMGU in chronic alcohol-fed rats and mice [14,15,28,118,119,120,121]. Chronic alcohol-fed mice also show whole-body insulin resistance, as assessed using an insulin tolerance test [100]. To the contrary, another study indicated that alcohol-fed mice were actually more insulin sensitive and that alcohol feeding could partially ameliorate high-fat diet-induced impairment in insulin action [90].

### 4.2. Hepatic Insulin Action and Resistance

The euglycemic hyperinsulinemic clamp can differentiate insulin action at the level of the liver and peripheral tissues (especially muscle) when combined with the infusion of radiolabeled or stable isotope-labeled glucose. In contrast to the ability of insulin to increase glucose uptake in striated muscle and fat (see following sections), insulin normally inhibits hepatic glucose production (HGP). Hence, in this experimental paradigm, hepatic insulin resistance is manifested as a decrease in insulin-induced suppression of endogenous HGP. Although acute alcohol did not produce hepatic insulin resistance in humans [115], rodents consistently exhibit impaired hepatic insulin sensitivity following both acute and chronic alcohol ingestion [14,28,120,122].

Numerous mechanisms have been proposed explaining how alcohol produces hepatic insulin resistance; however, there are few consistent findings when comparing between independent laboratories. For example, a decrease in high-affinity insulin binding (B_max_ and K_d_) and insulin internalization has been reported in cultured hepatocytes isolated from chronic alcohol-fed rats [49,123], while a reduction in binding of the insulin-like growth factors-I and -II has also been observed [124]. In contrast, short-term incubation of hepatocytes with alcohol did not alter insulin binding [49]. Overnight incubation of hepatocellular carcinoma (HCC) cells (*i.e*., FOCUS) with alcohol blunts the insulin-induced increase in the phosphorylation of the insulin receptor-β subunit, IRS-1 and AKT [125,126]. However, incubation of another HCC cell line (Huh-7) with alcohol did not affect upstream elements of the insulin signaling pathway despite reducing AKT phosphorylation [126,127]. Lastly, in the liver of alcohol-fed rats, no change in the phosphorylation of the insulin receptor, IRS-1 or PI3K was observed under basal conditions, although an exaggerated phosphorylation of each of these proteins occurred in response to exogenous insulin stimulation [120].

Presumably some of these and other inconsistencies in the data result from the use of different cell lines and animal models. For example, the severity of alcohol-induced hepatic insulin resistance is strain-dependent, being more pronounced in alcohol consuming Long-Evans compared to Sprague-Dawley rats [28]. This strain difference has the potential to provide mechanistic insight under *in vivo* conditions by allowing for the identification of differentially regulated signal transduction pathways central to glucose homeostasis. This approach has been used to reveal the relative importance of increased p53 protein abundance by activating TIGAR (Tp53-induced glycolysis and apoptosis regulator) and decreasing hepatic fructose-2,6-bisphosphate levels which mediate alcohol-induced hepatic insulin resistance and liver damage [28]. Additionally, the amount of alcohol consumed by animals may also contribute to the varied outcomes between models. In this regard, rats consuming a relatively low-dose (4 g/kg/day) of alcohol show evidence of enhanced insulin signaling via increased binding of PI3K with IRS-1 and the subsequent activation of AKT, whereas insulin signaling is impaired in rats consuming relatively high (13 g/kg/day) doses of alcohol [128]. In this latter and other subsequent work, the decreased AKT phosphorylation was posited to result from disrupted signaling following the alcohol-mediated induction of TRB3 (a mammalian homolog of *Drosophilia* tribbles-related protein 3) [128,129]. The hepatic insulin resistance may be related to the increased expression of PTEN (phosphatase tensin homologue deleted on chromosome 10) which dephosphorylates and inactivates PI3K in other models of alcohol intake [126].

### 4.3. Insulin Action and Resistance in Peripheral Tissues

#### 4.3.1. Skeletal Muscle Insulin Resistance

Skeletal muscle represents the largest body depot responsible for IMGU [130,131]. Therefore, an acute alcohol-induced decrease in IMGU by skeletal muscle per se has been inferred from experiments where whole-body insulin-stimulated glucose uptake is decreased during the glucose clamp (after correction for any residual endogenous HGP) [28,117,120]. Direct evidence for the suppression of muscle IMGU by acute alcohol was also reported in humans using the A-V difference method [52]. In further support, an alcohol-induced decrease in insulin-stimulated glucose disposal by skeletal muscle has been consistently detected in rats using radiolabeled 2-DG [12,14,117,118]. Interpretation of these seemingly consistent findings is complicated by a report showing that the magnitude of alcohol-induced insulin resistance is strain-dependent, with a more severe peripheral resistance observed in Sprague-Dawley rats compared to Long-Evans rats [14]. In contradistinction, as described above, the alcohol-induced hepatic insulin resistance is more prominent in Long-Evans *vs*. Sprague-Dawley rats. It has been suggested this strain difference may be related to differences in the generation of reactive oxygen species [28].

Glucose taken up by muscle can be oxidized via glycolysis or stored as glycogen (e.g., non-oxidative metabolism). Acute alcohol reduces insulin-stimulated non-oxidative glucose disposal into glycogen, as assessed by the incorporation of ^14^C-glucose, in a wide variety of fast- and slow-twitch muscles of rats [117]; however, in other studies this defect was only seen in fast-twitch muscles [65], which is the primary site of alcohol-induced myopathy [132]. Finally, in humans, alcohol acutely reduced glucose uptake and storage by muscle during a euglycemic hyperinsulinemic clamp, although this impaired glycogen deposition was not associated with reduced glycogen synthase activity [133].

Despite the consistent observation that acute and chronic alcohol impairs *in vivo*-determined IMGU by muscle, there is little consensus on the mechanism underlying the insulin resistance. Alcohol may theoretically blunt insulin action at a number of recognized regulatory steps including PI3K/AKT signal transduction and/or GLUT4 translocation [134]. For example, GLUT4 protein in the plasma membrane fraction of the gastrocnemius, but not in whole muscle homogenate, was reduced in alcohol consuming rats [14,57]. Similarly, *in vitro* incubation of differentiated myotubes with alcohol acutely inhibited insulin-stimulated GLUT4 translocation [70] and this response is dose-dependent [56]. Recruitment of GLUT4-containing vesicles to the cell membrane is dependent upon activation of AKT and the downstream phosphorylation of AS160 [135]. Although chronic alcohol consumption can antagonize insulin-stimulated AKT and AS160 phosphorylation in skeletal muscle of rats [14], other studies show phosphorylation of the insulin receptor, IRS-1, PI3K and AKT was not impaired (and may be increased) in muscle from alcohol-fed mice [120,136], suggesting a defect in signal transduction downstream of AKT. An alternative mechanism has been posited by Wan *et al.* [118] who reported that chronic alcohol feeding increases the mRNA and protein in muscle for the GTP-binding protein Gs-α, which in other conditions impairs IMGU [137].

The presence of peripheral insulin resistance in other catabolic states has been associated with the overproduction of the proinflammatory cytokines, tumor necrosis factor (TNF)-α or interleukin (IL)-6 [75,138]. It has been suggest that the alcohol-induced increase in TNFα and/or IL-6 in skeletal muscle in the basal state and the sustained elevation of these cytokines under hyperinsulinemic conditions is responsible for increased JNK (c-Jun N-terminal kinase) phosphorylation and consequently the phosphorylation of S307 IRS-1. These endpoints are increased in alcohol-fed mice under basal conditions [67], but it is unknown as to whether these alternations are maintained during a period of hyperinsulinemia. Several studies have reported increased TNFα and IL-6 in muscle from alcohol-fed animals [14,139,140] and these proinflammatory cytokines enhance both JNK phosphorylation and other stress-activated kinases [141]. Such data are consistent with the observation that muscle insulin resistance was only detected in Sprague-Dawley rats which exhibited a sustained elevation in both TNFα and IL-6 during basal and hyperinsulinemic conditions. IRS-1 is a potential down-stream target of JNK and elevated TNFα impairs insulin action via JNK-mediated Ser-phosphorylation of IRS-I [142]. Alcohol feeding relatively selectively blunts the insulin-induced increase in AKT and AS160 phosphorylation in Sprague-Dawley rats which is consistent with impairment of this putative signaling pathway. Collectively, data are consistent with an alcohol-induced reduction in GLUT4 translocation, at least in Sprague-Dawley rats.

#### 4.3.2. Cardiac Insulin Resistance

Data pertaining directly to the effect of alcohol on myocardial glucose uptake and insulin action are limited. Chronic alcohol consumption [14] but not acute alcohol administration [12], decreases IMGU in heart of Sprague-Dawley rats. It is unclear whether this differential effect is due to the different duration of alcohol exposure, the peak BAL achieved, or to another methodological constraint. This latter study indicated that for both basal and IMGU, the rank order (lowest to highest glucose uptake) for different myocardial regions was: atria < right ventricle < left ventricle. Further, the alcohol-induced decrease in myocardial IMGU was limited to ventricular tissue. Chronic alcohol feeding also impaired insulin-stimulated phosphorylation of the insulin receptor, IRS-1 and AKT in heart from alcohol-fed mice compared to pair-fed controls, in association with an increase in ER stress [68]. The generation of acetaldehyde may contribute to alcohol-induced insulin resistance in cardiac muscle as overexpression of ALDH2 in heart prevents some of the defects in insulin signal transduction and contractile function [67]. In contrast, exposure of H9c2 cardiomyocytes to alcohol *in vitro* increased glucose uptake and the translocation of GLUT4 to the plasma membrane, although alcohol exposure was extremely acute (30 min) and the duration of the effect was not assessed [70].

#### 4.3.3. Insulin Action in Adipose Tissue

In the sole study using acute alcohol administration, insulin-stimulated glucose uptake did not differ in adipocytes isolated from control and alcohol-treated rats [12]. In contrast, independent groups have reported relatively consistent data indicating that IMGU is decreased in several adipose depots (epididymal, subcutaneous and omental) of chronic alcohol-fed rats when determined either under *in vivo* [14,122] or *in vitro* [57,71,122] conditions. It is possible that this differential effect of alcohol on IMGU in adipose tissue results from differences in the duration of alcohol exposure the acute *in vitro* incubation of adipocytes with alcohol does not impair IMGU [143,144]. Chronic alcohol feeding also decreased *in vitro*-determined insulin-stimulated glucose uptake in isolated adipocytes [72] and this was associated with a failure of GLUT4 to fully translocate to the cell membrane, but was independent of the PI3K/AKT pathway [71]. The alcohol-induced decrease in IMGU in adipose tissue is consistent with the concomitant insulin-induced decrease in the AUC for plasma free fatty acid and glycerol concentrations [14,145], and may result because alcohol blunts the normal insulin-mediated suppression of lipolysis [6,72,145].

## 5. Alcohol and Diabetes

The extensive literature indicating the ability of acute and chronic alcohol intake to often times antagonize insulin-stimulated glucose disposal in peripheral tissues and the suppression of hepatic glucose production might be anticipated to exacerbate the development of type 2 diabetes mellitus. However, the consensus from a host of epidemiological studies is that moderate alcohol consumption is associated with reduced risk of type 2 diabetes, although in some studies the protective effect of alcohol was only observed in women [146,147,148,149]. Moderate alcohol consumption has been reported to decrease the risk of diabetes by approximately 30% [148]. It was only in the instances of prolonged heavy alcohol intake (e.g., ~60 g/day for men; 50 g/day for women) where alcohol had a deleterious effect [146,149]. Although the underlying mechanism for this protective effect remains equivocal, several studies have also reported a U- or J-shaped relationship between alcohol consumption and either insulin sensitivity or plasma insulin concentrations [24,109,150].

## 6. Conclusions

Although light to moderate alcohol consumption has potentially favorable effects on the cardiovascular system and potentially other tissues, excessive chronic alcohol consumption or acute intoxication adversely effects essentially all organs, ultimately increasing morbidity and mortality [151]. However, whether these adverse effects in humans are mediated by changes in glycemic control and/or insulin action is far from clear. As presented above there is considerable inconsistency in the literature for many of the specific areas related to glucose homeostasis, making general conclusions difficult. While some of the divergent results may stem from species differences (rat/mice *vs*. humans), there is also discordant results from studies within the same species. One pertinent caveat for all of the studies reported herein was the use preclinical animal models with little or no underlying pathology. While such models would be expected to decrease variability of the endpoints assessed, humans with longstanding alcohol abuse may have concomitant comorbid conditions which may cloud the translational relevance of data from such preclinical models. Hence, the impact of alcohol on glucose homeostasis in animal models, for example, fed a high-fat [72,90,92] or high cholesterol diet [152] or in the aged animal [139,153] might well differ from their younger and/or “normal” fed counterparts and potentially be more representative of humans consuming alcohol over a prolonged time frame. Additionally, humans chronically consuming alcohol often have some type of hypercholesterolemia, cardiovascular disease and/or heart dysfunction, and only recently have attempts been made to mimic this situation in animal models [152,154,155,156]. It is also noteworthy that essentially all preclinical models reported have used ethanol per se as the intoxicant, while subjects in the long-term human studies have consumed drinks containing variable types of alcohol (e.g., wine, beer, liquor) which may not necessarily elicit the same host response [152,157]. With methodological advances, future studies should be able to illuminate more subtle or nuanced effects of alcohol on cellular and molecular mechanisms of action which will improve understanding of how this drug exerts its influence at the organ and organism level. However, care must be exercise in the use of preclinical models, both animals and cell-based, to investigate mechanisms underlying phenomena which are actually observed in humans with alcohol use disorders. Ultimately, the importance of these alcohol-induced effects on insulin action and glucose homeostasis will need to be assessed in the context of whether they significantly alter the risk for the development of type 2 diabetes and other metabolic disturbances.
